# A Digital Smoking Cessation Program for Heavy Drinkers: Pilot Randomized Controlled Trial

**DOI:** 10.2196/formative.7570

**Published:** 2020-06-08

**Authors:** Christopher W Kahler, Amy M Cohn, Catherine Costantino, Benjamin A Toll, Nichea S Spillane, Amanda L Graham

**Affiliations:** 1 Department of Behavioral and Social Sciences Center for Alcohol and Addiction Studies Brown University School of Public Health Providence, RI United States; 2 University of Oklahoma Health Sciences Center Oklahoma City, OK United States; 3 Brown University School of Public Health Providence, RI United States; 4 Medical University of South Carolina Charleston, SC United States; 5 Department of Psychology University of Rhode Island South Kingston, RI United States; 6 Innovations Center Truth Initiative Washington, DC United States; 7 Georgetown University Medical Center Lombardi Comprehensive Cancer Center Washington, DC United States

**Keywords:** smoking cessation, alcohol drinking, internet, text messaging, therapy

## Abstract

**Background:**

Heavy drinking (HD) is far more common among smokers compared with nonsmokers and interferes with successful smoking cessation. Alcohol-focused smoking cessation interventions delivered by counselors have shown promise, but digital versions of these interventions—which could have far greater population reach—have not yet been tested.

**Objective:**

This pilot randomized controlled trial aimed to examine the feasibility, acceptability, and effect sizes of an automated digital smoking cessation program that specifically addresses HD using an interactive web-based intervention with an optional text messaging component.

**Methods:**

Participants (83/119, 69.7% female; 98/119, 82.4% white; mean age 38.0 years) were daily smokers recruited on the web from a free automated digital smoking cessation program (BecomeAnEX.org, *EX*) who met the criteria for HD: women drinking 8+ drinks/week or 4+ drinks on any day and men drinking 15+ drinks/week or 5+ drinks on any day. Participants were randomized to receive EX with standard content (EX-S) or an EX with additional content specific to HD (EX-HD). Outcomes were assessed by web-based surveys at 1 and 6 months.

**Results:**

Participants reported high satisfaction with the website and the optional text messaging component. Total engagement with both EX-S and EX-HD was modest, with participants visiting the website a median of 2 times, and 52.9% of the participants enrolled to receive text messages. Participants in both the conditions showed substantial, significant reductions in drinking across 6 months of follow-up, with no condition effects observed. Although smoking outcomes tended to favor EX-HD, the condition effects were small and nonsignificant. A significantly smaller proportion of participants in EX-HD reported having a lapse back to smoking when drinking alcohol (7/58, 16%) compared with those in EX-S (18/61, 41%; χ^2^_1_=6.2; *P*=.01).

**Conclusions:**

This is the first trial to examine a digital smoking cessation program tailored to HD smokers. The results provide some initial evidence that delivering such a program is feasible and may reduce the risk of alcohol-involved smoking lapses. However, increasing engagement in this and other web-based interventions is a crucial challenge to address in future work.

**Trial Registration:**

ClinicalTrials.gov NCT03068611; https://clinicaltrials.gov/ct2/show/NCT03068611

## Introduction

### Background

Cigarette smokers have substantially higher rates of alcohol consumption than nonsmokers [1,2], and both observational [3,4] and clinical studies [5-8] find that greater alcohol use predicts a reduced odds of smoking cessation. Alcohol use is a common smoking relapse precipitant [9,10], with over one-third of heavy drinkers reporting alcohol use at the time of a smoking lapse, often in combination with being around other people who are smoking and with experiencing positive affect [11,12]. Laboratory-based experiments show that alcohol has a direct pharmacologic effect on increasing the urge to smoke, which is beyond the effects of alcohol-related cues (ie, beyond the learned association between drinking and smoking) [13]. This increase in the urge to smoke accounts, in part, for alcohol’s effect on smoking. Heavy drinking (HD) is associated with a substantially greater risk of smoking lapse compared with moderate drinking [11]. Experimental research indicates that this effect of HD is not due to dose-dependent effects of alcohol on the urge to smoke and may instead reflect changes in regulatory control due to intoxication [13].

Given the deleterious impact of alcohol use on smoking cessation, a number of behavioral interventions have been developed specifically to address HD among HD smokers who are seeking smoking cessation treatment. In the first trial of its kind, Kahler et al [14] found that a smoking cessation treatment designed to motivate reduction of alcohol consumption and mitigate the risk of alcohol-involved smoking lapses resulted in significantly reduced alcohol consumption over 6 months, with small positive effects on smoking cessation compared with standard cessation treatment. Toll et al [15] incorporated a brief intervention to reduce alcohol use in HD smokers calling a state quitline, which resulted in significantly higher rates of smoking abstinence at 7 months compared with standard quitline counseling, with a trend toward reduced HD. In a pilot efficacy trial, Ames et al [16] found that an integrated smoking and alcohol intervention, compared with a smoking-only intervention, resulted in somewhat greater cigarette smoking abstinence and significantly reduced alcohol use among treatment completers. Finally, Correa-Fernandez et al [17] found that a combined alcohol and smoking intervention did not significantly improve smoking cessation outcomes compared with a smoking-only intervention but significantly reduced drinking in those who successfully quit smoking. Together, these studies highlight the potential value of interventions that simultaneously address smoking and HD and the positive public health impact such combined interventions may have if they have sufficient population reach.

Internet-based smoking cessation interventions have great potential to reduce smoking rates at the population level, given their demonstrated reach and effectiveness. In 2017, 36% of US smokers searched the web for information about quitting smoking [18], and hundreds of thousands register on web-based cessation programs each year [18,19]. Evidence-based internet interventions deliver the core components of cessation treatment through engaging, multimodal formats, oftentimes free of charge; are scalable and cost-efficient; and yield quit rates comparable with in-person and phone-based interventions [20,21]. However, across smoking cessation websites, alcohol is only minimally addressed [22,23]. Given that one-fourth to one-third of smokers seeking cessation services are heavy drinkers [5,24,25], enhancing internet-based smoking cessation interventions with alcohol-related content could have a substantial population impact.

### Objectives

Despite the potential promise and importance of addressing HD in smoking cessation treatment, models for incorporating an HD intervention into digital smoking cessation programs have not yet been developed. The objective of this study was to examine the feasibility and acceptability of a digital smoking cessation program that was developed to address HD specifically. HD smokers were randomly assigned to either a standard publicly available program or a modified version of the program that addressed HD in depth. Feasibility was assessed by recruitment rates, and acceptability was assessed by examining engagement and satisfaction with the elements of the program. Preliminary effect sizes were obtained on smoking and alcohol use outcomes at 1 and 6 months postrandomization with the hypothesis that addressing HD in depth would lead to less alcohol use and more smoking abstinence than the standard program. Reduced odds of alcohol-involved lapses were examined as a putative mechanism of action for the HD intervention.

## Methods

### Participants and Procedure

This pilot trial used a 2-group randomized control design. All procedures were approved by the Brown University Institutional Review Board, and the study was registered on ClinicalTrials.gov (NCT03068611). Participants were 119 newly registered users on BecomeAnEX.org (EX), a digital smoking cessation program developed and managed by Truth Initiative—a nonprofit public health organization in the United States dedicated to ending cigarette smoking (formerly the American Legacy Foundation). EX was developed in collaboration with the Mayo Clinic Nicotine Dependence Center aligning with the tobacco clinical practice guidelines [26] and first went on the web in 2008. Study recruitment for this project was conducted over 7 weeks (from May 10, 2017, to July 3, 2017).

New registered users on EX were eligible for the study if they met the following inclusion criteria: (1) current daily smoker, (2) met the National Institute on Alcohol Abuse and Alcoholism (NIAAA) criteria for the past month (8+ drinks/week or 4+ drinks on ≥1 occasion in the past month for women and 15+ drinks/week or 5+ drinks on ≥1 occasion in the past month for men), (3) ≥18 years old, (4) willing to provide contact information, (5) no prior use of the EX website, and (6) US residence based on Internet Protocol (IP) address. Individuals were ineligible if they reported a history of severe alcohol withdrawal symptoms because we did not wish to recommend abstaining from alcohol to participants for whom unsupervised alcohol withdrawal could be dangerous.

All procedures were conducted on the web. Recruitment information asked new registered users about their interest in participating in a research study *to develop and test a version of BecomeAnEX that is designed specifically for smokers who drink alcohol*. Interested individuals, then, completed the study eligibility screening questions. Those screened as eligible then read and electronically signed a web-based informed consent document that described study procedures, including the saliva collection procedure at follow-ups for those reporting smoking abstinence. Next, they verified their email address and completed the baseline survey. Individuals who were ineligible, who did not complete the consent, or who did not verify their email were directed to the standard EX (EX-S) program but were not enrolled in the study. Participants were compensated US $20 via an Amazon gift card code for their time completing the baseline survey.

#### Randomization

Eligible participants were assigned on a 1:1 basis by a computer algorithm to either EX with standard content (EX-S) or EX with additional content specific to HD (EX-HD). To ensure a balance of potentially key background factors, block randomization was conducted within 8 blocks formed by a 2 × 2 × 2 matrix of the following factors: sex (male and female), age (≤30 years old and >30 years old), and frequency of HD (<weekly and ≥weekly). The age cutoff of 30 years was chosen to ensure that both conditions had a very similar number of participants who would be classified as young adults, for whom the use of mobile technology and the context of alcohol consumption may be different when compared with older adults. Those randomized had access only to their respective treatment condition.

#### Follow-Ups

At 1 and 6 months postrandomization, all participants were sent an email to complete a follow-up survey. Those who did not complete the survey within 10 days of the initial email were called by study staff. Participants were compensated US $30 for the 1-month and US $50 for the 6-month follow-up via an Amazon gift card code.

### Intervention Content

#### Website

Primary elements of the EX-S site are summarized in Table 1. A *My Quit Plan* page displayed a checklist for users that showed if each of the site’s core components had been completed and provided the recommended next steps. The EX-HD website included all elements of EX-S and additional pages addressing alcohol use (Table 1), aligning with our previous counselor-delivered interventions [14,15] and including content mirroring and linking to NIAAA’s *Rethinking Drinking* website [27]. A *Managing Alcohol Use* tab was added to *My Quit Plan* in EX-HD so that participants could view and print their alcohol change plan. Participants were given no specific guidance about how often to use the website, consistent with how EX is used by the public. Participants had access to the website throughout their participation in the study and retained that access as registered users after study participation concluded.

**Table 1:** Website elements.

Element^a^		Description
**Elements in EX-Standard**		
	(1) Quit Date	A tool for participants to select a quit smoking date, which is recommended to occur within 2 weeks from registration.
	(2) Cigarette Tracker	An interactive exercise to help participants identify the time of day and triggers for smoking by tracking cigarette use.
	(3) Beat Your Smoking Triggers	An interactive exercise to help participants formulate strategies to dissociate cigarettes from smoking triggers.
	(4) Build Your Support System	An interactive exercise for participants to identify helpful supporters to improve their efforts to quit as well as unsupportive people in their life they may need to avoid while quitting.
	(5) Choose a Quit Smoking Aid	A strategic planning exercise where participants indicate their plan for pharmacotherapy use.
	(6) EX Community	A large web-based social network of current and former smokers.
	(7) Educational Content	Didactic, multimodal content that addresses strategies to prepare for quit day, cope with slips, and prevent relapse. It includes videos about tobacco addiction and pharmacotherapy.
**Additional elements in EX-Heavy Drinker**		
	(1) Alcohol and Quitting Smoking	A content page on the risk of smoking relapse associated with heavy alcohol use.
	(2) What’s Your Drinking Pattern	An interactive exercise that provides personalized normative feedback on drinking using gender-based national US drinking norms.
	(3) Risks of Heavy Drinking	Content page on NIAAA^b^ guidelines for low-risk drinking and interactive effects of smoking and HD^c^ on head and neck cancer risk.
	(4) Managing Drinking	An interactive exercise for participants to evaluate the importance of changing drinking while quitting smoking and articulate their own reasons for change.
	(5) Benefits of Changing Drinking	An interactive exercise where participants endorse which benefits they might experience if they stopped or cut down on drinking.
	(6) Make a Plan to Manage Drinking	An interactive exercise for setting goals regarding drinking during the quit smoking attempt (eg, stop drinking for a specified time after quitting and set a maximum daily alcohol consumption limit) and making plans to minimize the risk of smoking when drinking (eg, drinking only with people who do not smoke or in places where smoking is not allowed).
	(7) Strategies for Limiting Drinking	An interactive exercise where participants select strategies they could use for limiting drinking such as practicing drink refusal skills and self-monitoring drinking.
	(8) Alcohol and Staying Quit	A content page highlighting the ongoing risk of smoking relapse associated with drinking alcohol.

^a^Participants were randomized to either EX with standard content (EX-S) or EX with additional content specific to HD (EX-HD). Those who were randomized to EX-HD received all EX-S content as well as the EX-HD content. Individuals received only the intervention web pages to which they were randomized each time they logged into the website.

^b^NIAAA: National Institute on Alcohol Abuse and Alcoholism.

^c^HD: heavy drinking.

#### Text Messaging

The EX text message program used was consistent with empirically supported text messaging smoking cessation programs in both frequency of texting and duration of the program [28]. Users signed up for text messaging during registration and had to reply *OK* to an initial message to complete text message enrollment; they could unenroll at any time by texting *STOP*. Messages encouraged interaction with the program through yes/no, true/false, and multiple-choice questions and provided quitting advice, positive reinforcement, reminders to avoid smoking triggers, information about nicotine and nicotine withdrawal, and tailored support around a set quit date.

Text messaging was the same intensity in both conditions. Participants received 2 messages/day before their selected quit date, 3-5 messages/day for 2 weeks starting on their quit date, and then 1-2 messages/day through 6 weeks past the quit date; the maximum number of messages they could receive was 128. Only 1 EX-S text message directly addressed alcohol use. In the EX-HD program, 24 of the EX-S messages (18.8%) were replaced with an alcohol-focused message. These texts were developed through an iterative process that involved extracting key content from previous alcohol-focused smoking interventions [12,14,15] and adapting it for a short messaging format consistent with the messages used in the EX-S text messaging program. Messages were piloted with a sample of 12 participants who gave feedback on content and tone. These texts provided information about the effect of HD on health and quitting smoking (eg, “For smokers who drink regularly, more than 35% of slips back to smoking happen when they are drinking alcohol”), encouraged and reinforced reductions in drinking (eg, “Great! You’re showing your commitment to your health; consider the benefits of drinking less and record them here”), provided links to alcohol-focused content on EX-HD, and reminded participants to anticipate situations in which they might drink (eg, “Any events coming up this week where you might be around alcohol? Reply with Y or N”).

### Measures

#### Baseline Data

At baseline, demographic information was collected about sex, age, race/ethnicity, education, employment, income, and marital status. Cigarette dependence was assessed using the Fagerström Test for Cigarette Dependence [29], and the perceived importance of quitting smoking was assessed with a single-item scale (0=*not at all important* to 10=*extremely important*) [14]. Severity of alcohol problem was assessed at baseline using the short inventory of problems [30]. Participants were asked about the importance of cutting down or stopping drinking while quitting smoking using a single-item scale (0=*not at all important* to 10=*extremely important*) and if they planned to cut down or stop drinking while quitting smoking (0=*no*, 1=*possibly*, 2=*probably*, and 3=*definitely*) [14].

#### Website Utilization

Utilization data from the 6 months following randomization were extracted from the EX database, including number of website visits and pages viewed. Utilization of EX-S content was assessed by tracking if participants completed each of the 7 core EX activities, and utilization of EX-HD content was assessed by tracking completion of the 8 HD activities.

#### Satisfaction With and Perceptions of the Program

At 1 month, satisfaction with the overall program was assessed using the 8-item client satisfaction questionnaire (CSQ-8) [31], which assesses satisfaction with provided services on a 1 to 4 scale, where higher scores indicate higher levels of satisfaction (alpha=.92). In addition, all participants provided single-item ratings of specific aspects of the program, including rating their *overall experience* with the website (1=*poor* to 5=*excellent*) and the extent to which the website made them consider how alcohol might affect their quitting (1=*Did not use*, 2=*Not at all*, 3=*A little*, 4=*Moderately*, and 5=*Very much/a lot*). Those who enrolled in text messaging were asked about their *overall experience* with the text messaging program (1=*poor* to 5=*excellent*) and if it helped them quit smoking and manage their drinking, respectively, (from 1=*completely disagree* to 5=*strongly agree*). They also rated if the number of text messages on alcohol was 1=*too few*, 2=*just right*, or 3=*too many*.

#### Smoking Outcomes

At baseline and follow-up, cigarette use was assessed using a daily report of smoking behavior over the past 7 days [32]. The primary smoking outcome was a self-reported 7-day abstinence from smoking cigarettes and other combustible tobacco products at 1 and 6 months [33]. Continuous abstinence was a secondary smoking outcome, defined as having made a quit attempt with no reported slips at both the 1- and 6-month follow-ups [33]. Participants reporting smoking abstinence at 6 months were invited to provide a saliva sample by mail via a Salimetrics collection kit to biochemically verify smoking status [34,35]. Participants were paid US $25 for returning the kit within 48 hours of receipt. Abstinence was confirmed with a cotinine concentration of <15 ng/mL.

#### Alcohol Outcomes

At baseline and follow-up, participants reported past 30-day drinking, including the number of days they drank 4+ drinks (for women)/5+ drinks (for men), and provided a daily report of drinking over the past 7 days [32]. The primary alcohol outcome was the number of HD days in the past 30 days at 1 and 6 months, with the 30-day window chosen to capture variability in this more uncommon behavior. The secondary outcome was the total number of drinks consumed in the past 7 days at those follow-ups. Although this 7-day assessment window is narrower than that used for inclusion in the study, this secondary outcome may provide a better estimate of total drinking as it is based on day-level reporting rather than using a quantity-frequency estimate.

#### Smoking Lapses

A series of questions were used to assess the perceptions of the causes of initial smoking lapses following previous work in the area [11,12]. At follow-up, participants were asked if they had made a quit attempt since enrolling. Those reporting a quit attempt were asked if they had smoked since that attempt, and if so, “What was the major cause of your smoking?,” where *drinking alcohol* was 1 of the 10 response options, including *don’t know* and *other*. Those selecting *drinking alcohol* were classified as having an alcohol-involved lapse.

### Analysis Plan

All analyses were conducted using SAS 9.4 [36]. We first examined the number of participants passing study milestones (eg, screening, consent, baseline, and follow-up) and the baseline characteristics of the sample. The acceptability of EX-HD was examined by comparing EX-S and EX-HD on website and text message utilization and program satisfaction using *t* tests and nonparametric tests. In exploratory analyses, we also examined if baseline characteristics predicted engagement metrics. The preliminary efficacy of EX-HD was examined by comparing smoking and alcohol use outcomes using *t* tests and chi-square tests. We used chi-square analyses to test if EX-HD reduced the odds of alcohol-involved smoking lapses compared with EX-S. In exploratory analyses, we examined if engagement metrics were associated with perceptions of the program. We also conducted generalized estimating equations (GEE) covarying age, sex, and baseline frequency of HD (ie, the variables included in the randomization scheme) to examine if treatment effects across 1 and 6 months were moderated by gender, motivation to change (ie, perceived importance of quitting smoking and reducing drinking, respectively), level of website engagement, and text messaging enrollment. An alpha level of .05 was used for all analyses, without adjustment for multiple testing, because the primary purpose of the analyses was to identify patterns of results that might inform future modifications and improvement of the EX-HD program.

## Results

### Feasibility

**Figure 1:**
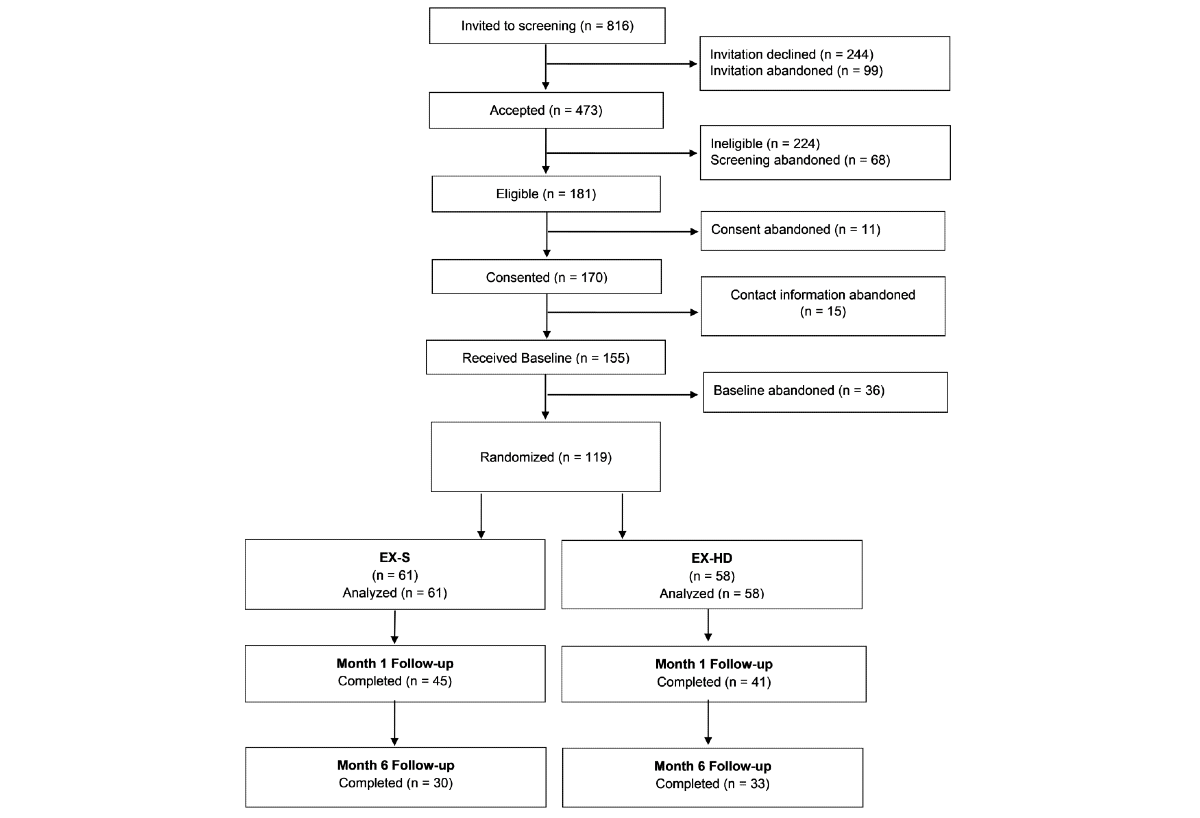
A Consolidated Standards of Reporting Trials diagram showing participant flow. EX-HD: BecomeAnEX with additional content specific to heavy drinking; EX-S: BecomeAnEX with standard content.

Figure 1 shows the Consolidated Standards of Reporting Trials diagram of participant flow through study milestones. During the recruitment period, there were a total of 816 newly registered EX users with US IP addresses, who each received an invitation to participate in the study. Of these, 57.9% (473/816) completed screening, and 38.3% of those were screened as eligible. A third (34.3%) of participants who were screened as eligible were not enrolled in the study because they did not complete the consent process, did not confirm their contact information via email, or did not complete the baseline survey, leaving 119 enrolled participants, with 61 randomized to EX-S and 58 to EX-HD. Overall, 14.6% of all new US users of EX during the recruitment window participated in the study.

Table 2 shows the demographic and clinical characteristics of the enrolled participants overall and by condition. Follow-up rates were 72.3% at 1 month and 52.9% at 6 months. There were no significant differences in demographics, baseline clinical variables, or intervention condition between follow-up completers and noncompleters. At the 6-month follow-up, 14% (17/119) of participants reported 7-day abstinence from smoking and nicotine and were sent a saliva collection kit. Of these, 42% (7/17) returned the kit, with 4 having abstinence confirmed by testing. Three participants reported smoking in the past week at the time they returned the sample, of whom 2 were over the cutoff of 15 ng/mL.

**Table 2:** Baseline demographic and clinical characteristics for the entire sample and by treatment condition.

Variable			Overall (N=119)		EX-S^a^ (n=61)		EX-HD^b^ (n=58)
Female gender, n (%)			83 (69.7)		42 (69)		41 (71)
Age (years), mean (SD)			38.0 (11.8)		38.6 (12.9)		37.1 (10.6)
**Race, n (%)**							
	White	98 (82.4)		50 (82)		48 (83)	
	Black/African American	13 (10.9)		6 (10)		7 (12)	
	Native American	1 (0.8)		0 (0)		1 (2)	
	Asian	0 (0.0)		0 (0)		0 (0)	
	Pacific Islander	0 (0.0)		0 (0)		0 (0)	
	Multiple races	6 (5.0)		5 (8)		1 (2)	
	Other	1 (0.8)		0 (0)		1 2)	
	Hispanic/Latino	4 (3.4)		1 (2)		3 (5)	
**Education, n (%)**							
	Less than high school	7 (5.9)		4 (7)		3 (5)	
	High school	20 (16.8)		8 (13)		12 (21)	
	Some college/tech/business	66 (55.5)		37 (61)		29 (50)	
	College graduate or higher	26 (21.8)		12 (20)		14 (24)	
**Employment status, n (%)**							
	Unemployed	21 (17.6)		9 (15)		12 (21)	
	Employed part/full time	84 (70.6)		48 (79)		36 (62)	
	Other^c^	14 (11.8)		4 (7)		10 (17)	
**Income, n (%)**							
	Less than US $30,000	52 (43.7)		28 (46)		24 (41)	
	US $30,000-US $59,999	32 (26.9)		18 (29)		14 (24)	
	US $60,000-US $89,999	18 (15.1)		6 (10)		12 (21)	
	US $90,000 or more	17 (14.3)		9 (15)		8 (14)	
**Marital status, n (%)**							
	Married or cohabiting	47 (39.5)		26 (43)		21 (36)	
	Single (never married)	36 (30.3)		17 (28)		19 (33)	
	Widow/separated/divorced	36 (30.3)		18 (29)		18 (31)	
**Clinical characteristics, mean (SD)**							
	Cigarettes per day in past 7 days	17.1 (9.1)		15.9 (74)		18.4 (10.5)	
	FTCD^d^ total score	5.1 (2.3)		4.9 (2.3)		5.3 (2.3)	
	SIP^e^ total score	9.8 (10.1)		10.1 (9.7)		9.5 (10.6)	
	Number of HD^f^ days^g^	6.9 (6.8)		7.3 (7.2)		6.6 (6.4)	
	Number of drinks in the past 7 days	16.0 (12.8)		14.2 (11.7)		17.8 (13.7)	
	Importance of quitting smoking^h^	9.1 (1.6)		9.2 (1.5)		9.1 (1.6)	
	Importance of reducing drinking^h^	6.5 (3.6)		7.1 (3.5)		5.9 (3.6)	
**Intent to change drinking,^i^ n (%)**							
	No	9 (7.6)		3 (5)		6 (10)	
	Possibly	29 (24.4)		13 (21)		16 (28)	
	Probably	33 (27.7)		16 (26)		17 (29)	
	Definitely	48 (40.3)		29 (47)		19 (33)	

^a^EX-S: EX-standard.

^b^EX-HD: EX-heavy drinker.

^c^Includes students, homemakers, and retired individuals.

^d^FTCD: Fagerström Test for Cigarette Dependence.

^e^SIP: short inventory of problems.

^f^HD: heavy drinking.

^g^In the 30 days before study enrollment.

^h^Single-item rating from 0=not important to 10=extremely important.

^i^Participants were asked if they planned to cut down or stop drinking while quitting smoking.

### Website Engagement and Acceptability

Website engagement, as operationalized by site visits, total page views, pages viewed per visit, total minutes on the site, and completion of EX-S activities are shown in Table 3 and were similar by condition. Just under half of the participants (48.7%) completed <1 EX activity. Just over two-thirds (42/61, 69%) of the participants set a quit date at least once through the website, with 13% (8/61) setting a quit date more than once. The median time spent on the site was 8 min in both conditions, with just over a quarter of participants spending more than 25 min on the site.

Among those assigned to EX-HD, 74% (43/58) of participants did not complete any EX-HD activities and 12% (7/58) completed more than half (Table 3). Exploratory analyses of the clinical factors in Table 2 that might be related to engagement with EX-HD indicated that 35% (6/17) of those who reported they would *probably* change their drinking completed more than half of EX-HD exercises compared with 0% (0/6), 6% (1/16), and 0% (0/19) of those reporting *no*, *possibly*, or *definitely*, respectively (χ^2^_3_=12.6; *P*=.006). Other smoking and drinking characteristics were not significantly associated with high EX-HD website engagement.

With respect to acceptability, participants in both the conditions rated the overall experience with the website very similarly, with means between *good* and *very good* (Table 3). Contrary to expectations, participants in the both conditions provided similar ratings about how much the website made them consider the impact of alcohol on their quit efforts, with means near the anchor of *moderately*. In an exploratory analysis within the EX-HD condition, ratings on this item were significantly higher among those who had completed at least one EX-HD activity (mean 4.67, SD 0.71) compared with those who had not (mean 3.58, SD 1.39; t_38_=2.25; *P*=.03).

**Table 3:** Website engagement and acceptability.

Variable		Experimental group		*P* value^c^	
		EX-S^a^ (control)	EX-HD^b^		
Sample size, n		61	58		N/A^d^
Number of visits, median (IQR)		2 (1-3)	2 (1-3)		.47
Number of pages viewed, median (IQR)		17 (6-31)	13 (4-33)		.48
Pages viewed per visit, median (IQR)		6.7 (3.3-11.3)	5.6 (3.0-11.3)		.49
Minutes on website, median (IQR)		8.1 (3.3-25.9)	8.1 (1.3-27.7)		.86
**EX-S activities completed, n (%)**					
	Set a quit date	42 (69)	39 (67)		.85
**Number of activities completed, n (%)**				.80	
	No exercises	13 (21)	15 (26)		
	1 exercise	17 (28)	13 (22)		
	2 exercises	15 (25)	15 (26)		
	3 exercises	8 (13)	6 (10)		
	4+ exercises	8 (13)	9 (15)		
**EX-HD activities completed, n (%)**					
	No exercises	N/A	43 (74)		N/A
	1 exercise	N/A	5 (9)		N/A
	2 exercises	N/A	1 (2)		N/A
	3 exercises	N/A	2 (3)		N/A
	4+ exercises	N/A	7 (12)		N/A
**Self-reported website satisfaction**					
	Total respondents, n (%)	45 (74)	40 (69)		N/A
	Overall experience, mean (SD)^e^	3.87 (0.97)	3.75 (0.95)		.58
	Caused reflection on alcohol use^f^, mean (SD)	3.91 (1.14)	3.82 (1.33)		.75

^a^EX-S: EX-standard.

^b^EX-HD: EX-heavy drinker.

^c^*P* values were determined using *t* tests for continuous variables, chi-square tests for categorical variables, and Wilcoxon rank sum tests for non-normal variables.

^d^N/A: not applicable.

^e^Response scale is 1-5 (1=Poor, 2=Fair, 3=Good, 4=Very Good, and 5=Excellent).

^f^Response scale 1-5 (1=Did not use, 2=Not at all, 3=A little, 4=Moderately, and 5=Very much/a lot).

### Text Messaging Engagement and Acceptability

Table 4 also shows text messaging engagement and acceptability. With respect to text messaging engagement, participants in EX-HD were significantly more likely to unenroll from text messaging compared with those in EX-S. However, the number of messages sent or received did not differ significantly by condition. Participants in EX-S and EX-HD rated the overall experience with the texting program very similarly, with means between *good* and *very good*. Likewise, they rated the helpfulness of the program very similarly. With regard to whether the text program helped them manage drinking while quitting smoking, those in EX-HD rated this item numerically higher (closer to the *somewhat agree* anchor), although this difference was not statistically significant. Regarding the number of alcohol-focused texts, participants in EX-HD rated these as slightly over *just right*, whereas those in EX-S rated them as slightly under *just right*, a difference which was significant.

**Table 4:** Text messaging engagement and acceptability.

Variable		Experimental group		*P* value^c^
		EX-S^a^ (control)	EX-HD^b^	
**Text message enrollment, n (%)**				.46
	Never signed up	16 (26)	16 (28)	
	Signed up/no initiation	15 (25)	9 (16)	
	Fully enrolled	30 (49)	33 (57)	
Unenrollment rate, n (%)		3 (13)^d^	14 (42)^e^	.01
**Messaging frequency, median (IQR)^f^**				
	Messages received by participant	116 (36-142)	86 (27-153)	.95
	Messages sent from participant	13 (3-26)	9 (3-14)	.47
**Text messaging acceptability**				
	Total respondents, n (%)	25 (41)	22 (38)	N/A^g^
	Overall experience, mean (SD)^h^	3.64 (1.00)	3.86 (1.25)	.90
	Helpful with quitting smoking,^i^ mean (SD)	3.84 (1.10)	3.86 (1.13)	.94
	Helpful with managing drinking,^i^ mean (SD)	2.96 (1.34)	3.64 (1.14)	.07
	Number of alcohol-related messages,^j^ mean (SD)	1.89 (0.67)	2.32 (0.57)	.02

^a^EX-S: EX-standard.

^b^EX-HD: EX-heavy drinker.

^c^*P* values were determined using t tests for continuous variables, chi-square tests for categorical variables, and Wilcoxon rank sum tests for non-normal variables.

^d^N=30.

^e^N=33.

^f^Of participants who were fully enrolled in the text messaging program, including those who were unenrolled.

^g^N/A: not applicable.

^h^Response scale is 1-5 (1=Poor, 2=Fair, 3=Good, 4=Very Good, 5=Excellent).

^i^Response scale is 1-5 (1=Completely disagree, 2=Somewhat disagree, 3=Neither agree nor disagree, 4=Somewhat agree, 5=Strongly agree).

^j^Response scale is 1-3 (1=Too few, 2=Just right, 3=Too many).

### Overall Program Satisfaction

CSQ-8 ratings were very similar between EX-S (mean 3.14, SD 0.59) and EX-HD (mean 3.07, SD 0.63; t_83_=0.57; *P*=.57).

### Alcohol Use Outcomes

Primary drinking outcomes (means and SDs) and between-group effect sizes are shown in Table 5. The number of drinks per week and percent HD days showed significant reductions from baseline to both 1-month and 6-month follow-up, regardless of condition (*P*s<.05). However, there was no clear pattern of differential reductions in drinking by condition, and between-group effect sizes were small.

**Table 5:** Summary of alcohol use and smoking outcomes at baseline and at each follow-up.

Variables			EX-S^a^ (n=61)	EX-HD^b^ (n=58)	Effect size^c^	*P* value
**Drinking outcomes^d^, mean (SD)**						
	**Number of HD^e^** **days in the past 30 days**					
		Baseline (EX-S=61, EX-HD=58)	7.3 (7.2)	6.6 (6.4)	−0.01	.94
		1 month (EX-S=45, EX-HD=40)	2.9 (4.7)	4.4 (6.3)	0.14	.36
		6 months (EX-S=30, EX-HD=33)	2.6 (3.5)	3.8 (6.7)	0.03	.92
	**Total number of drinks in the past 7 days^f^**					
		Baseline (EX-S=61, EX-HD=58)	14.2 (11.7)	17.8 (13.7)	0.26	.16
		1 month (EX-S=45, EX-HD=41)	10.6 (12.1)	8.8 (10.1)	−0.23	.29
		6 months (EX-S=30, EX-HD=33)	8.1 (8.2)	7.7 (10.1)	−0.14	.57
**Smoking outcomes^g^**						
	**Smoking abstinence,** **n (%)**					
		1 month (past 7 days)	10 (16)	10 (17)	0.02	.90
		6 months (past 7 days)	9 (15)	11 (19)	0.11	.54
		6 months—biochemically verified	1 (2)	3 (5)	0.20	.36^h^
		Continuous abstinence^i^	2 (3)	5 (9)	0.23	.22^h^
	**Smoking lapse behavior,** **n (%)**					
		Reporting any lapse^j^	77 (89)	73 (84)	−0.14	.51
		Reporting alcohol-involved lapse^j^	18 (41)	7 (16)	−0.56	.01

^a^EX-S: EX-standard.

^b^EX-HD: EX-heavy drinker.

^c^Effect size is expressed as Cohen *d* for continuous data and Cohen *h* for dichotomous outcomes.

^d^*P* values and effect sizes are calculated using *t* tests on log-transformed data due to positive skewness and kurtosis.

^e^HD: heavy drinking.

^f^Obtained from a 7-day timeline follow-up.

^g^Denominator is all 119 participants with those participants with missing data on smoking status considered as smoking. Abstinence was defined as self-reported abstinence from all combustible tobacco products.

^h^*P* values were calculated using Fisher exact tests rather than chi-square tests due to small cell sizes.

^i^Continuous abstinence was defined as those who made a quit attempt and reported no slips for 6 months.

^j^Denominator was 87 participants who reported having made a quit attempt at either the 1-month or 6-month follow-up.

### Smoking Outcomes

Smoking outcomes are shown in Table 5. The effect sizes were small and not statistically significant.

### Alcohol-Involved Lapses

A total of 90 participants (EX-S: n=46 and EX-HD: n=44) completed a 1-month or 6-month smoking lapse survey. Of those 90, 87 (97%) made a quit smoking attempt since enrollment. Although the percentage of participants reporting a lapse was similar across conditions (Table 4), EX-HD participants were significantly less likely to report having an alcohol-involved lapse.

### Moderators, Engagement, and Outcomes: Exploratory Analyses

#### Moderators

For all smoking and alcohol use outcomes reported above, exploratory analyses indicated no significant interactions between gender and treatment condition, *P*s>.45. Likewise, neither perceived importance of quitting smoking nor perceived importance of cutting down on drinking while quitting smoking interacted with treatment condition significantly, *P*s>.45.

#### Engagement

Due to the non-normal distribution of website utilization metrics (ie, site visits, pages viewed, and EX-S activities completed), we dichotomized each using a median split and summed them to create a 0-3 ordinal scale, where higher scores reflected greater engagement. Website engagement was not significantly correlated with overall program satisfaction as assessed by the CSQ-8 (*r*_85_=0.16; *P*=.15). In GEE models, greater website engagement was associated with significantly higher odds of smoking abstinence (odds ratio 1.72, 95% CI 1.20-2.49; *P*=.003) but was not significantly associated with either drinking outcome. Text messaging enrollment was not significantly correlated with website engagement (*r*_119_=0.10; *P*=.29), or with overall program satisfaction (*r*_85_=0.07; *P*=.55). Text messaging enrollment was not significantly associated with smoking or drinking outcomes, and the effect of treatment conditions on smoking and drinking outcomes did not differ based on the level of website engagement or text messaging enrollment, *P*s>.40.

## Discussion

### Principal Findings

This pilot randomized controlled trial was built on a well-established evidence-based smoking cessation website to address HD in the context of smoking cessation, incorporating key elements of our previously tested person-delivered approaches for addressing HD in smoking cessation. The findings provided strong support for the feasibility of recruiting participants who are HD smokers enrolled in a publicly available digital smoking cessation program. Almost 60% of invited individuals agreed to be screened, a rate higher than that in a previous study from this website [37]. In general, registrants on EX who elect to be screened for research tend to be heavier smokers and more motivated to quit compared with those who do not screen [37]. Of those who screened, almost 40% were eligible, with the vast majority subsequently enrolled in the trial; such screening and enrollment rates compare favorably with other studies on web-based smoking cessation programs [38,39]. Participants recruited from EX tended to be female, although HD is more common in men [4]. This gender imbalance likely reflects that women are more likely to look for health information on the web [40]. There was a rapid rate of recruitment in this trial, with 119 participants successfully enrolled in only 7 weeks. This is notable given that HD smokers have represented a small minority of participants in other clinical trials of web-based cessation interventions [39] and have been largely ignored in cessation studies. The results demonstrate that it would be feasible to conduct a large-scale trial of EX-HD recruiting solely from BecomeAnEX.

Follow-up rates ranged from just over 70% at 1 month to just over 50% at 6 months, similar to other trials of web-based smoking cessation programs [21], where the risk of bias is relatively low given roughly equal follow-up rates by condition. Additional methods to enhance completion of follow-up assessment, such as offering an additional bonus incentive for survey completion within 24 hours [39] or providing more frequent brief communications from the study between follow-up visits, would be important to utilize in a future trial. Consistent with prior research [41,42], there were large discrepancies between self-reported and biochemically verified smoking abstinence due to missing data rather than misreported smoking status. We agree with previous recommendations on the verification of smoking status, which suggest that using biochemical verification in large-scale web-based cessation trials is of limited value and is not feasible [43].

Overall, satisfaction with website content was high regardless of the treatment condition. However, although about two-thirds of participants set a quit date over the web, engagement with the website was low, with a median of 2 visits to the website, consistent with 2 large-scale trials on internet cessation, one of which was recruited from BecomeAnEX [39,44]. The lack of differences by condition in website engagement suggests that the additional alcohol content in EX-HD did not hinder engagement. However, only one-quarter of the participants in EX-HD completed a web-based activity specific to EX-HD. Given that website engagement was related to better smoking outcomes across conditions—echoing a recent study among smokers from EX with an alcohol use disorder [45]—testing ways to increase exposure to and engagement with website content remains a high priority. Identifying strategies to boost engagement with digital health behavior change programs has been noted as a priority for the field [46]. Tailoring the website experience to users’ preferences may be one means of enhancing engagement. Indeed, among those assigned to EX-HD, exploratory analyses indicated that those who were strongly considering—but not definitely—changing their drinking while quitting smoking were the most likely to engage with the alcohol-related content. Future iterations of EX-HD could assess interest in changing drinking in an initial assessment and then provide more or less alcohol content based on user intentions. Such tailored and dynamic interventions may enhance engagement. Regardless of further efforts to enhance program engagement, future clinical trials of EX-HD should determine sample size requirements, accounting for the fact that only one-quarter of participants will have meaningful interaction with the intervention content, substantially reducing the potential effect size for EX-HD.

About half of the participants were enrolled in text messaging. Those in EX-HD were more likely to unenroll from text messaging. EX-HD participants tended to want fewer alcohol-focused messages, and those in EX-S tended to want more, which could account for the greater rate of unenrollment by EX-HD participants. Participants receiving text messaging in EX-HD, compared with those in EX-S, rated the messaging program as more helpful for managing drinking, although the effect did not reach the .05 significance level. Future text messaging interventions will need to balance the potential for overmessaging on alcohol use while ensuring that texts address alcohol in sufficient depth, perhaps by allowing some user choice over content. Again, allowing for user tailoring of text messaging content and frequency may obviate concerns about oversaturating users with content that they do not consider relevant. In this trial, participants signed up for text messaging before they knew that they would participate in a study of alcohol-focused intervention, and therefore, they did not sign up knowing that they would receive a substantial number of alcohol-related texts. Given that almost 1 in 5 messages were alcohol-focused, a more optimum proportion would likely be lower than that and could be tailored based on user preference.

EX-HD did not result in greater reductions in drinking compared with EX-S. However, reductions in drinking seen in both conditions in this study were larger than those reported in most cessation trials [12,14,17,47], on the order of about 50% reductions in both HD days and drinks per week. Such changes could represent regression to the mean given that participants had to meet certain thresholds for drinking immediately before participation. However, they also could reflect the fact that over two-thirds of participants reported that they probably or definitely intended to cut down on drinking when trying to quit smoking. Before receiving either intervention, participants believed that changing their drinking habits when quitting smoking was quite important, with a mean of over 6 on a 0-10 measure of importance.

Although reductions in drinking were similar across conditions, EX-HD resulted in significantly reduced odds of alcohol-related smoking lapses compared with EX-S, mirroring results of an initial trial testing an in-person smoking cessation program targeting HD smokers [14]. These outcomes may reflect a heightened awareness of the risk of alcohol-related smoking lapses caused by either web or text messaging content. That this effect emerged was somewhat surprising, given the low exposure to alcohol-related content overall. Given that smoking lapses for HD smokers often occur in the context of drinking, such an effect may be important in improving smoking outcomes.

For all smoking outcomes, effect sizes were small, and none approached significance. Smoking outcomes were similar to those reported in a recent trial of smokers from EX [48], with abstinence rates ranging from 15% to 19% at 1- and 6-month follow-ups. The relatively small effect sizes align with those found in a study of quitline counseling with HD smokers, which showed a statistically significant 3.2% difference that favored the HD intervention [15]. A small effect size in the context of a website that reaches thousands of smokers each month could have a meaningful impact on public health with no added costs beyond the website development.

### Limitations

The primary limitations of this pilot study include its modest sample size and follow-up rates, which make it inappropriate to draw conclusions about its efficacy and potential impact. The sample size also limits the degree of depth to which we can understand factors that might have impacted engagement and satisfaction with EX-HD content. When examining factors that might relate, for example, to the utilization of text messaging, the number of participants who opted into text messaging (n=63) makes in-depth analyses impractical. Likewise, because there was modest engagement in the website content, it was not possible to examine experimental outcomes among those who had substantial contact with intervention content. The lack of biochemical validation of the primary smoking outcomes is also a limitation that is common in many trials of web-based cessation programs.

Recruitment was done from the available pool of newly registered users of EX. Therefore, the sample was limited to those with computer literacy and access and reflected the demographics of EX users, who tend to be white women with relatively high education, demographics that are roughly consistent with other web-based cessation trials [20]. A more diverse sample could be achieved in a full-scale randomized controlled trial of EX-HD by deactivating the recruitment of overrepresented groups once targets for those groups are reached [37]. In addition, publicity for digital smoking cessation programs may need to be targeted to websites and social media outlets where minority smokers are more represented. Engaging smokers from underrepresented groups in the development of campaigns to promote digital cessation programs is likely to improve the reach of these programs and is an important area for future research and practice.

### Conclusions

HD has been highlighted as an important predictor of smoking relapse and cessation failure, but it is rarely addressed in web-based smoking cessation platforms. As such, enhancing proven web-based cessation interventions with alcohol-related content is critical to leveraging the potential public health impact of a broad reach treatment modality for heavy drinkers who smoke. In our study, many new HD registrants to a digital smoking cessation program are willing to participate in a trial focused on smoking cessation targeting HD. EX-HD is acceptable and may be useful in reducing alcohol-involved smoking lapses. The feasibility and acceptability of EX-HD, coupled with a modest initial indication of its clinical promise, warrants testing its effects on both drinking and smoking outcomes in a fully powered large-scale clinical trial. A critical next step in intervention development efforts is to focus on methods for increasing exposure to and engagement with intervention content, including tailoring user experiences to their interest and intention in changing drinking.

## Appendix

Multimedia Appendix 1 CONSORT EHEALTH checklist (v.1.6.1).

## Abbreviations

CSQ-8: client satisfaction questionnaire
EX: BecomeAnEX.org
EX-HD: EX with additional content specific to HD
EX-S: EX with standard content
GEE: generalized estimating equations
HD: heavy drinking
IP: Internet Protocol
