# Supplementary material for: A Digital Smoking Cessation Program for Heavy Drinkers: Pilot Randomized Controlled Trial
Source: JMIR Form Res. 2020 Jun 8;4(6):e7570. doi: 10.2196/formative.7570 (PMC7308890; doi:10.2196/formative.7570)
Supplement: Multimedia Appendix 1 [file formative_v4i6e7570_app1.pdf]

CONSORT-EHEALTH Checklist V1.6.2 Report

Manuscript Number 7570

(based on CONSORT-EHEALTH V1.6), available at [http://tinyurl.com/consort-ehealth-v1-6].

Date completed

8/21/2019 18:13:13

by

Christopher Kahler

Digital smoking cessation for heavy drinkers: A pilot randomized controlled trial

TITLE

1a-i) Identify the mode of delivery in the title

Digital smoking cessation for heavy drinkers: A pilot randomized controlled trial

1a-ii) Non-web-based components or important co-interventions in title

"DIGITAL"

1a-iii) Primary condition or target group in the title

"smoking cessation for heavy drinkers"

ABSTRACT

1b-i) Key features/functionalities/components of the intervention and comparator in the METHODS section of the ABSTRACT

"This pilot randomized controlled trial examined feasibility, acceptability, and effect sizes for a digital smoking cessation program that specifically addressed heavy drinking (HD) using a web-based intervention with an optional text messaging component. " "Participants were randomized to receive standard EX content (EX-S) or a version of EX that included content specific to HD (EX-HD)."

1b-ii) Level of human involvement in the METHODS section of the ABSTRACT

"automated digital smoking cessation program "

1b-iii) Open vs. closed, web-based (self-assessment) vs. face-to-face assessments in the METHODS section of the ABSTRACT

"Outcomes were assessed by web-based surveys at 1 and 6 months."

1b-iv) RESULTS section in abstract must contain use data

"Participants (N = 119; 69.7% female; 82.4% white)" "A significantly smaller proportion of participants in EX-HD reported having a lapse back to smoking when drinking alcohol (16.3%) compared to those in EX-S (40.9%), □2(1) = 6.22, P = .013."

1b-v) CONCLUSIONS/DISCUSSION in abstract for negative trials

"This pilot trial provides some evidence that delivering a digital cessation intervention tailored to HD smokers is feasible and may reduce the risk of alcohol-involved smoking lapses. However, the effects of the intervention are likely to be limited by low rates of engagement with intervention content, a crucial challenge to address in future work in this area."

INTRODUCTION

2a-i) Problem and the type of system/solution

" Cigarette smokers have substantially higher rates of alcohol consumption than nonsmokers [1-3], and both observational [4-6] and clinical studies [7-12] find that greater alcohol use predicts a reduced odds of smoking cessation. Alcohol use is a common smoking relapse precipitant [13, 14] with over one-third of heavy drinkers reporting alcohol use at the time of a smoking lapse [15, 16]. Given the deleterious impact of alcohol use on smoking cessation, a number of behavioral interventions have been developed specifically to address heavy drinking (HD) among HD smokers who are seeking smoking cessation treatment."

2a-ii) Scientific background, rationale: What is known about the (type of) system

"In the first trial of its kind, Kahler et al. [17] found that a smoking cessation treatment designed to motivate reductions in alcohol consumption and mitigate the risk of alcohol-involved smoking lapses resulted in significantly reduced alcohol consumption over 6 months, with small positive effects on smoking cessation compared to standard cessation treatment. Toll et al. [18] incorporated brief intervention to reduce alcohol use in HD smokers calling a state quitline, which resulted in significantly higher rates of smoking abstinence at 7 months compared to standard quitline counseling, with a trend towards reduced HD. In a pilot efficacy trial, Ames et al. [19] found that integrated smoking and alcohol intervention, compared to a smoking-only intervention, resulted in somewhat greater cigarette smoking abstinence and significantly reduced alcohol use among treatment completers. Finally, Correa-Fernandez et al. [20] found that combined alcohol and smoking intervention did not significantly improve smoking cessation outcomes compared to smoking-only intervention but significantly reduced drinking in those who successfully quit smoking. Together, these studies highlight the potential value of interventions that simultaneously address smoking and HD and the positive public health impact such combined interventions may have if they have sufficient population reach. "

Does your paper address CONSORT subitem 2b?

"This study examined the feasibility and acceptability of a digital smoking cessation program that specifically addressed HD. HD smokers were randomly assigned to either a standard program or a version that addressed HD in depth. Feasibility was assessed by recruitment rates, and acceptability was assessed by examining engagement and satisfaction with the elements of the program. Preliminary effect sizes were obtained on smoking and alcohol use outcomes at 1 and 6 months post-randomization with the hypothesis that addressing HD in depth would lead to less alcohol use and more smoking abstinence than a standard program. Reduced odds of alcohol-involved lapses was examined as a potential mechanism of action for the HD intervention. "

METHODS

3a) CONSORT: Description of trial design (such as parallel, factorial) including allocation ratio

"This trial used a 2-group randomized control design." "Eligible participants were assigned on a 1:1 basis by computer algorithm to either standard EX content (EX-S) or a version of EX that specifically targeted HD (EX-HD). Block randomization was conducted within 8 blocks formed by a 2 X 2 X 2 matrix of the following factors: sex (male, female), age (<30 years old, >30 years old), and frequency of HD (< weekly, > weekly). Those randomized had access only to their respective treatment condition. "

3b) CONSORT: Important changes to methods after trial commencement (such as eligibility criteria), with reasons

None

3b-i) Bug fixes, Downtimes, Content Changes

Did not arise.

4a) CONSORT: Eligibility criteria for participants

"New registered users on EX were eligible for the study if they met the following inclusion criteria: 1) current daily smoker; 2) met NIAAA criteria for past month HD (8+ drinks/week or 4+ drinks on > 1 occasion in the past month for women; 15+ drinks/week or 5+ drinks on > 1 occasion in the past month for men); 3) > 18 years old; 4) willing to provide contact information; 5) no prior use of the EX website; and 6) US residence based on IP address. Individuals were ineligible if they reported a history of severe alcohol withdrawal symptoms, because we did not wish to recommend abstaining from alcohol to participants for whom unsupervised alcohol withdrawal could be dangerous."

4a-i) Computer / Internet literacy

"Finally, the use of web-based recruitment meant that the sample was limited to those with computer literacy and access."

4a-ii) Open vs. closed, web-based vs. face-to-face assessments:

The methods describe that assessments were conducted online.

4a-iii) Information giving during recruitment

At the end of website registration, all users were asked about their interest in participating in a research study "to develop and test a version of BecomeAnEX that is designed specifically for smokers who drink alcohol."

4b) CONSORT: Settings and locations where the data were collected

Participants were recruited only from online users of BecomeAnEX.org

4b-i) Report if outcomes were (self-)assessed through online questionnaires

"At 1 and 6 months post-randomization, all participants were sent an email to complete a follow-up survey. Those who did not complete the survey within 10 days of the initial email were called by study staff."

4b-ii) Report how institutional affiliations are displayed

Participants knew that they were signing up for BecomeAnEX and were told it was a Brown University study

5) CONSORT: Describe the interventions for each group with sufficient details to allow replication, including how and when they were actually administered

5-i) Mention names, credential, affiliations of the developers, sponsors, and owners

"This project was supported by a grant from the National Institute on Alcohol Abuse and Alcoholism, R34AA024593. Benjamin Toll testifies as an expert witness on behalf of plaintiffs who have filed litigation against the tobacco companies. Amanda Graham is employed by Truth Initiative, which licenses an enterprise version of BecomeAnEX to employers, health plans, and other tobacco control organizations."

5-ii) Describe the history/development process

The program did undergo some initial testing with EX users. However, the changes from that were modest and this process was not central to the pilot RCT itself.

5-iii) Revisions and updating

None.

5-iv) Quality assurance methods

None.		
**5-v) Ensure replicability by publishing the source code, and/or providing screenshots/screen-capture video, and/or providing flowcharts of the algorithms used**		
We have a document that document the flow of web pages and one that includes all text messages sent, which can be made available on request.		
**5-vi) Digital preservation**		
This is not possible since BecomeAnEX does not archive older versions of the site. This is something we would prioritize in future collaborations.		
**5-vii) Access**		
Participants access the site at home or on their phone.		
**5-viii) Mode of delivery, features/functionalities/components of the intervention and comparator, and the theoretical framework**		
See Table 1		
**5-ix) Describe use parameters**		
Used ad libitum		
**5-x) Clarify the level of human involvement**		
No human involvement needed.		
**5-xi) Report any prompts/reminders used**		
"Text messaging was the same intensity in both conditions. Participants received 2 messages/day prior to their selected quit date, 3-5 messages/day for 2 weeks starting on their quit date and then 1-2 message/day through 6 weeks past quit date. Only one standard EX text message directly addressed alcohol use. In the EX-HD program, twenty-four of the standard EX messages were replaced with an alcohol-focused message. These texts were developed through an iterative process which involved extracting key content from prior alcohol-focused smoking interventions [16-18] and adapting it for short messaging format consistent with the messages used in the standard EX text messaging program. These texts provided information about the effect of HD on health and quitting smoking, encouraged and reinforced reductions in drinking, provided links to alcohol-focused content on EX-HD, and reminded participants to anticipate situations in which they might drink. "		
**5-xii) Describe any co-interventions (incl. training/support)**		
None		
**6a) CONSORT: Completely defined pre-specified primary and secondary outcome measures, including how and when they were assessed**		
"Smoking outcomes. At baseline and follow-ups, cigarette use was assessed using a daily report of smoking behavior over the past 7 days [40]. The primary smoking outcome was self-reported 7-day abstinence from smoking cigarettes and other combustible tobacco products at 1 and 6 months. Continuous abstinence was a secondary smoking outcome, defined as having made a quit attempt with no reported slips and no tobacco smoking in the past 7 days at both 1 month and 6 months. Participants reporting smoking abstinence at 6 months were invited to provide a saliva sample by mail via a Salimetrics© collection kit to biochemically verify smoking status [41, 42]. Participants were paid $25 for returning the kit within 48 hours of receipt. Abstinence was confirmed with a cotinine concentration under 15 ng/ml.<br>Alcohol outcomes. At baseline and follow-ups, participants reported past 30-day drinking, including number of days they drank 4+ drinks (for women)/5+ drinks (for men), and provided a daily report of drinking over the past 7 days [40]. The primary alcohol outcome was number of HD days in the past 30 days at 1 and 6 months, with the 30-day window chosen to capture variability in this more uncommon behavior. The secondary outcome was the total number of drinks consumed in the past 7 days at those follow-ups; although this 7-day assessment window is narrower than that used for inclusion in the study, this secondary outcome may provide a better estimate of total drinking since is based on day-level reporting rather than using a quantity-frequency estimate.<br>Smoking lapses. A series of questions was used to assess the context of initial smoking lapses following prior work in the area [15, 16]. At follow-ups, participants were asked if they had made a quit attempt since enrolling. Those reporting a quit attempt were asked whether they had smoked since that attempt and if so, whether they were drinking alcohol at the time of that initial lapse (i.e., whether they had an alcohol-involved lapse)."		
**6a-i) Online questionnaires: describe if they were validated for online use and apply CHERRIES items to describe how the questionnaires were designed/deployed**		
No.		
**6a-ii) Describe whether and how "use" (including intensity of use/dosage) was defined/measured/monitored**		
Table 3 provides all of the metrics of use		
**6a-iii) Describe whether, how, and when qualitative feedback from participants was obtained**		
None		
**6b) CONSORT: Any changes to trial outcomes after the trial commenced, with reasons**		
Participants were recruited only from online users of BecomeAnEX.org		
**7a) CONSORT: How sample size was determined**		
**7a-i) Describe whether and how expected attrition was taken into account when calculating the sample size**		
Treatment development guidelines typically consider Stage 1a work to involve about 15 participants and small-scale Stage 1b pilot trials to have 30-60 participants. We believe that we needed to double these numbers because of the nature of the population studied. Not all participants enrolling will set a quit date or have significant interaction with the website. In addition, follow-up rates for web-based cessation trials are likely to be in the 70-75% range, which is lower than would typically be achieved in many pilot RCTs where enrollment, assessment and counseling often occur face-to-face.Therefore, a sample of N=120 for should give us more adequate numbers of participants who engage with the site and who use features such as text messaging. Our primary objective will be to determine whether EX-HD shows promise, rather than to determine statistical significance between groups at certain p values. Relatively small effect sizes for a web-based intervention may still have clinical importance given how many people can be reached with this modality and that the cost of running and maintaining EX-HD would not differ from the cost of EX once it is fully developed. In our trial with telephone counseling, a significant difference in smoking abstinence rates of 3% between condition was obtained with a sample of just under 2000 participants. We could readily achieve a sample of that size in a future trial with BecomeAnEX.		
**7b) CONSORT: When applicable, explanation of any interim analyses and stopping guidelines**		
"Smoking outcomes. At baseline and follow-ups, cigarette use was assessed using a daily report of smoking behavior over the past 7 days [40]. The primary smoking outcome was self-reported 7-day abstinence from smoking cigarettes and other combustible tobacco products at 1 and 6 months. Continuous abstinence was a secondary smoking outcome, defined as having made a quit attempt with no reported slips and no tobacco smoking in the past 7 days at both 1 month and 6 months. Participants reporting smoking abstinence at 6 months were invited to provide a saliva sample by mail via a Salimetrics© collection kit to biochemically verify smoking status [41, 42]. Participants were paid $25 for returning the kit within 48 hours of receipt. Abstinence was confirmed with a cotinine concentration under 15 ng/ml.<br>Alcohol outcomes. At baseline and follow-ups, participants reported past 30-day drinking, including number of days they drank 4+ drinks (for women)/5+ drinks (for men), and provided a daily report of drinking over the past 7 days [40]. The primary alcohol outcome was number of HD days in the past 30 days at 1 and 6 months, with the 30-day window chosen to capture variability in this more uncommon behavior. The secondary outcome was the total number of drinks consumed in the past 7 days at those follow-ups; although this 7-day assessment window is narrower than that used for inclusion in the study, this secondary outcome may provide a better estimate of total drinking since is based on day-level reporting rather than using a quantity-frequency estimate.<br>Smoking lapses. A series of questions was used to assess the context of initial smoking lapses following prior work in the area [15, 16]. At follow-ups, participants were asked if they had made a quit attempt since enrolling. Those reporting a quit attempt were asked whether they had smoked since that attempt and if so, whether they were drinking alcohol at the time of that initial lapse (i.e., whether they had an alcohol-involved lapse)."		
**8a) CONSORT: Method used to generate the random allocation sequence**		
Eligible participants were assigned on a 1:1 basis by computer algorithm to either standard EX content (EX-S) or a version of EX that specifically targeted HD (EX-HD). Block randomization was conducted within 8 blocks formed by a 2 X 2 X 2 matrix of the following factors: sex (male, female), age (<30 years old, >30 years old), and frequency of HD (< weekly, > weekly).		
**8b) CONSORT: Type of randomisation; details of any restriction (such as blocking and block size)**		
Eligible participants were assigned on a 1:1 basis by computer algorithm to either standard EX content (EX-S) or a version of EX that specifically targeted HD (EX-HD). Block randomization was conducted within 8 blocks formed by a 2 X 2 X 2 matrix of the following factors: sex (male, female), age (<30 years old, >30 years old), and frequency of HD (< weekly, > weekly).		
**9) CONSORT: Mechanism used to implement the random allocation sequence (such as sequentially numbered containers), describing any steps taken to conceal the sequence until interventions were assigned**		
Eligible participants were assigned on a 1:1 basis by computer algorithm to either standard EX content (EX-S) or a version of EX that specifically targeted HD (EX-HD). Block randomization was conducted within 8 blocks formed by a 2 X 2 X 2 matrix of the following factors: sex (male, female), age (<30 years old, >30 years old), and frequency of HD (< weekly, > weekly).		
**10) CONSORT: Who generated the random allocation sequence, who enrolled participants, and who assigned participants to interventions**		
This was automated. A staff member put together the sequences ahead of time.		
**11a) CONSORT: Blinding - If done, who was blinded after assignment to interventions (for example, participants, care providers, those assessing outcomes) and how**		
**11a-i) Specify who was blinded, and who wasn't**		
Participants were not blinded but outcome assessors (when needed) were.		
**11a-ii) Discuss e.g., whether participants knew which intervention was the "intervention of interest" and which one was the "comparator"**		
Participants did not receive any information about what the "standard" EX would look like and therefore were not specifically informed that they were receiving the intervention of interest.		
**11b) CONSORT: If relevant, description of the similarity of interventions**		

"Text messaging was the same intensity in both conditions. Participants received 2 messages/day prior to their selected quit date, 3-5 messages/day for 2 weeks starting on their quit date and then 1-2 message/day through 6 weeks past quit date. Only one standard EX text message directly addressed alcohol use. In the EX-HD program, twenty-four of the standard EX messages were replaced with an alcohol-focused message. These texts were developed through an iterative process which involved extracting key content from prior alcohol-focused smoking interventions [16-18] and adapting it for short messaging format consistent with the messages used in the standard EX text messaging program. These texts provided information about the effect of HD on health and quitting smoking, encouraged and reinforced reductions in drinking, provided links to alcohol-focused content on EX-HD, and reminded participants to anticipate situations in which they might drink. "<br>"Website. Primary elements of EX-S site are summarized in Table 1. A My Quit Plan page displayed a checklist for users that showed whether each of the site's core components had been completed and provided recommended next steps. The EX-HD website included all elements of EX-S and additional pages addressing alcohol use (Table 1), aligning with our prior counselor-delivered interventions [17, 18]. Normative feedback on drinking, descriptions of the risks of HD, goal setting and strategies for limiting drinking mirrored pages on these topics on NIAAA's Rethinking Drinking website (https://www.rethinkingdrinking.niaaa.nih.gov). EX-HD also included links to Rethinking Drinking pages. A Managing Alcohol Use tab was added to My Quit Plan in EX-HD so participants could view and print their alcohol change plan."		
**12a) CONSORT: Statistical methods used to compare groups for primary and secondary outcomes**		
We first examined the number of participants passing study milestones (e.g., screening, consent, baseline, follow-up) and the baseline characteristics of the sample. Acceptability of EX-HD was examined by comparing EX-S and EX-HD on website and text message utilization and program satisfaction using t-tests and non-parametric tests. Preliminary efficacy of EX-HD was examined by comparing smoking and alcohol use outcomes using t-tests and chi-square tests. To account for the repeated assessment of alcohol and smoking outcomes over time and variables included in the randomization scheme, we conducted negative binomial and logistic regression analyses using generalized estimating equations (GEE) that covaried age, sex, and baseline frequency of HD. We used chi-square analyses to test whether EX-HD reduced the odds of alcohol-involved smoking lapses compared to EX-S and whether lower odds of alcohol-involved lapses was associated with better smoking outcomes at 1 and 6 months. Exploratory analyses examined whether treatment effects were moderated by gender and motivation to change (i.e., perceived importance of quitting smoking and of reducing drinking, respectively) and whether the effect of EX-HD differed based on level of website engagement and text messaging enrollment.		
**12a-i) Imputation techniques to deal with attrition / missing values**		
Missing data imputation was not done beyond the standard assumption of missing = smoking. In a pilot trial, the ability to conduct imputation is greatly limited.		
**12b) CONSORT: Methods for additional analyses, such as subgroup analyses and adjusted analyses**		
"Exploratory analyses examined whether treatment effects were moderated by gender and motivation to change (i.e., perceived importance of quitting smoking and of reducing drinking, respectively) and whether the effect of EX-HD differed based on level of website engagement and text messaging enrollment."		
RESULTS		
**13a) CONSORT: For each group, the numbers of participants who were randomly assigned, received intended treatment, and were analysed for the primary outcome**		
See Figure 1		
**13b) CONSORT: For each group, losses and exclusions after randomisation, together with reasons**		
See Figure 1		
**13b-i) Attrition diagram**		
See Figure 1		
**14a) CONSORT: Dates defining the periods of recruitment and follow-up**		
"Study recruitment was conducted over 7 weeks (May to July, 2018). "		
**14a-i) Indicate if critical "secular events" fell into the study period**		
None		
**14b) CONSORT: Why the trial ended or was stopped (early)**		
Not applicable		
**15) CONSORT: A table showing baseline demographic and clinical characteristics for each group**		
Table 2		
**15-i) Report demographics associated with digital divide issues**		
Table 2		
**16a) CONSORT: For each group, number of participants (denominator) included in each analysis and whether the analysis was by original assigned groups**		
**16-i) Report multiple "denominators" and provide definitions**		
Table 4		
**16-ii) Primary analysis should be intent-to-treat**		
Table 4 and analysis plan.		
**17a) CONSORT: For each primary and secondary outcome, results for each group, and the estimated effect size and its precision (such as 95% confidence interval)**		
We provide 95% CI for each outcome in the GEE analysis along with raw data.		
**17a-i) Presentation of process outcomes such as metrics of use and intensity of use**		
Table 3		
**17b) CONSORT: For binary outcomes, presentation of both absolute and relative effect sizes is recommended**		
Table 3 and Results section		
**18) CONSORT: Results of any other analyses performed, including subgroup analyses and adjusted analyses, distinguishing pre-specified from exploratory**		
". Exploratory analyses examined whether treatment effects were moderated by gender and motivation to change (i.e., perceived importance of quitting smoking and of reducing drinking, respectively) and whether the effect of EX-HD differed based on level of website engagement and text messaging enrollment."		
**18-i) Subgroup analysis of comparing only users**		
Not done.		
**19) CONSORT: All important harms or unintended effects in each group**		
None to report		
**19-i) Include privacy breaches, technical problems**		
**19-ii) Include qualitative feedback from participants or observations from staff/researchers**		
None to report		
DISCUSSION		
**20) CONSORT: Trial limitations, addressing sources of potential bias, imprecision, multiplicity of analyses**		
**20-i) Typical limitations in ehealth trials**		
"The primary limitations of this pilot study include its modest samples size and follow-up rates that make it inappropriate to draw conclusions about its efficacy and potential impact. The sample size also limits the degree of depth to which we can understand factors that might have impacted engagement and satisfaction with EX-HD content. When examining factors that might related, for example, to utilization of text messaging, the numbers of participants who opted into text messaging (n = 63) makes in-depth analyses impractical. Likewise, because there was modest engagement in the website content, it was not possible to examine experimental outcomes among those who had substantial contact with intervention content. Lack of biochemical validation of the primary smoking outcomes is also a limitation common with many trials of online cessation programs. Finally, the use of web-based recruitment meant that the sample was limited to those with computer literacy and access."		
**21) CONSORT: Generalisability (external validity, applicability) of the trial findings**		
**21-i) Generalizability to other populations**		
**21-ii) Discuss if there were elements in the RCT that would be different in a routine application setting**		
**22) CONSORT: Interpretation consistent with results, balancing benefits and harms, and considering other relevant evidence**		
**22-i) Restate study questions and summarize the answers suggested by the data, starting with primary outcomes and process outcomes (use)**		
"This pilot randomized controlled trial provided strong support for the feasibility of recruiting participants who are HD smokers enrolled in a publicly-available digital smoking cessation program. " "Overall, satisfaction with website content was high regardless of treatment condition. However, even though about two-thirds of participants set a quit date online, engagement with the website was modest, with a median of two visits to the website."		
**22-ii) Highlight unanswered new questions, suggest future research**		
"The feasibility and acceptability of EX-HD, coupled with modest initial indication of its clinical promise, warrant testing its effects on both drinking and smoking outcomes in a fully powered large-scale clinical trial. Future intervention development efforts should focus on methods for increasing exposure to and engagement with intervention content including tailoring user experiences to their interest and intention in changing drinking."		
Other information		
**23) CONSORT: Registration number and name of trial registry**		

"Clinical Trial Registration Number: # NCT03068611"		
**24) CONSORT: Where the full trial protocol can be accessed, if available**		
The protocol will be available on NCT when that is released. The NCT # is provided.		
**25) CONSORT: Sources of funding and other support (such as supply of drugs), role of funders**		
"This project was supported by a grant from the National Institute on Alcohol Abuse and Alcoholism, R34AA024593"		
**X26-i) Comment on ethics committee approval**		
"All procedures were approved by the Brown University IRB"		
**x26-ii) Outline informed consent procedures**		
**X26-iii) Safety and security procedures**		
**X27-i) State the relation of the study team towards the system being evaluated**		
"Amanda Graham is employed by Truth Initiative, which licenses an enterprise version of BecomeAnEX to employers, health plans, and other tobacco control organizations. "		
